# Medical school ranking and provider outpatient Medicare Part D claims for antibiotics among older patients in the USA

**DOI:** 10.1093/jacamr/dlae191

**Published:** 2024-11-23

**Authors:** Mayar Al Mohajer, David Slusky, David Nix, Catia Nicodemo

**Affiliations:** Department of Medicine, Baylor College of Medicine, Houston, TX, USA; Department of Continuous Education, University of Oxford, Oxford, UK; Department of Primary Health Care, University of Oxford, Oxford, UK; Department of Economics, University of Kansas, Lawrence, KS, USA; National Bureau of Economic Research, Cambridge, MA, USA; IZA—Institute of Labor Economics, Bonn, Germany; Department of Pharmacy Practice and Science, University of Arizona, Tucson, AZ, USA; Department of Continuous Education, University of Oxford, Oxford, UK; Department of Primary Health Care, University of Oxford, Oxford, UK; Department of Economics, University of Verona, Verona, Italy

## Abstract

**Background:**

Our study aimed to assess whether there was a relationship between graduating from higher-ranked medical schools and the rate of prescribing antibiotics among Medicare Part D providers in the USA.

**Methods:**

The study obtained data from the Medicare Part D Prescribers (FY2013-2021) and the Doctor and Clinicians National repositories. A regression model was fitted to assess the relationship between provider medical school ranking and the rate of antibiotic days supplied per 100 beneficiaries at the provider level.

**Results:**

A total of 197 540 providers were included. No association was found between the medical school ranking and the rate of antibiotics days supplied per 100 beneficiaries. Instead, the type of provider is associated with the prescription rates. Hospitalists and Emergency Medicine providers had fewer days supplied per 100 beneficiaries than Family Medicine providers. In contrast, students, more experienced providers (>20 years since medical school graduation) and females had more days supplied per 100 beneficiaries.

**Conclusion:**

Our study highlights the need for robust outpatient stewardship interventions and incorporating an outcome-based approach to antibiotic stewardship curricula in medical and mid-level provider schools.

## Introduction

Excessive antimicrobial utilization is associated with antibiotic resistance, *Clostridioides difficile* colitis, and increased morbidity and mortality.^1^ The CDC estimated that over 2.8 million people develop antibiotic-resistant bacteria, and 223 000 develop *C. difficile* colitis, resulting in over 47 000 deaths combined. By 2050, it is projected that over 10 million individuals will die from infections caused by resistant bacteria.^2^

Patients aged 65 years and older are particularly vulnerable to sequelae or resistance and are 50% more likely to receive antimicrobials than younger individuals. Evidence shows that outpatient antibiotic overprescription in older patients varies by region and provider specialty. A CDC study involving Medicare Part D beneficiaries (about 70% of all Medicare patients) found that while only 36% of all prescribers are located in the US South, this region accounted for 48% of the highest volume prescribers (top 10% of total prescribers). Similarly, Family Medicine (FM) and Internal Medicine (IM) providers represent only 25% of all prescribers, yet they made up 52% of the highest volume prescribers. This indicates a significant overrepresentation of both Southern providers and these two specialties among the top prescribers, highlighting regional and specialty-specific differences in antibiotic prescribing.^3^

Understanding the behavioural reasons for differences in antibiotic prescription among providers is crucial for tackling overprescription. Three systematic reviews^4–6^ examined the role of provider differences in antibiotic use. They showed that inequities in antibiotic use could be explained by provider age, gender, years of practice, experience (e.g. mid-level), country of medical school and specialty.

In addition to these provider-level differences, the variability in antimicrobial stewardship (AS) training across US medical schools may contribute to disparities in prescribing behaviours. While some schools have adopted comprehensive, evidence-based stewardship curricula, others offer limited instruction, leading to potential gaps in clinical practice.^7,8^ This underscores the need to evaluate how educational outcomes impact prescribing behaviours, as these discrepancies may further influence overall prescribing patterns.

For example, Schnell *et al*.^9^ found lower opioid prescription rates among graduates of higher-ranked medical schools compared with lower-ranked schools. However, the impact of medical school ranking on provider antimicrobial utilization was not previously assessed in the literature. Investigating this unexplored area is needed to evaluate whether a higher medical school ranking indicates better training on antibiotic stewardship.

Given the existing gap in the literature, our study aims to analyse the effect of general practitioners’ graduate school ranking on their outpatient Medicare Part D claims for antibiotics in the USA. We hypothesize that providers who graduated from higher-ranked medical schools would have lower antibiotic claims than those from lower-ranked schools.

## Study data and methods

### Data

In this study, five repositories of data were utilized: (i) the Medicare Part D Prescribers by provider data for fiscal years 2013–2021; (ii) the Medicare Part D Prescribers by provider and drug data for fiscal years 2013–2021; (iii) the Doctor and Clinicians National dataset (2017–2023); (iv) the 2023 teaching hospital repository; and (v) the 2023–2024 US News Best Medical School for Research.

The first repository (Medicare Part D Prescribers by provider)^10^ contained the characteristics of providers, medication claims and their patients. Provider variables include national provider identification (NPI), gender, specialty, office address and metropolitan area. Beneficiary variables comprised demographics (average age, counts of gender, race/ethnicity), dual public insurance (with Medicaid) and average risk score at the provider level.

The CMS Part D Prescribers by provider and drug datasets for FY2013–2021 (Repository 2)^11^ comprised detailed information on drugs prescribed (generic name, brand name, total claims, days supplied, number of 30-day refills and cost). The Doctor and Clinicians National dataset for FY2023 (Repository 3)^12^ contained medical education information for all providers. It included the NPI, provider name, credentials [Doctor of Medicine (MD)/Doctor of Osteopathic Medicine (DO)/Nurse Practitioner (NP) and Physician Assistant (PA)], medical school, graduation year and address. The Teaching Hospital set (Repository 4)^13^ lists US teaching hospitals and their addresses. The 2023–2024 US News Best Medical School for Research (Repository 5)^14^ ranks 192 medical and osteopathic US schools based on research activity.

### Inclusion and exclusion criteria

Providers were included if their specialty was FM, IM, Emergency Medicine (EM), Hospitalist, PA, NP or Student. Due to smaller populations, providers from US territories or overseas were excluded. Claims provided for topical antibiotics were not included, given their limited impact on antibiotic resistance.

Since CMS suppressed claims and beneficiary data of fewer than 11 for privacy reasons, an imputed value of five was assigned to the suppressed values to prevent underestimating true values. Providers with missing values for other variables were excluded. Sensitivity analysis was applied using imputed values of 1 and 9. It was also done for FY2013 and FY2019 [the first and last prior to the Coronavirus Disease 2019 (COVID-19 pandemic)].

### Provider education

The US News and World Report Ranking for Best Medical Schools for Research was used as a proxy for provider education. Providers were grouped into International Medical Graduates (IMG), US MDs ((1, 35), [35, 58), [58, 85), [85,118] and unranked), DOs (ranked and unranked) and mid-level practitioners (ranked and unranked).

### Study outcomes

The study’s main outcome was antibiotic days supplied per 100 beneficiaries. Secondary outcomes included antibiotic claims per 100 beneficiaries, days per claim and antibiotic cost per 100 beneficiaries.

### Covariates

The study controlled the following variables: fiscal year, provider gender, specialty, experience, teaching location, metropolitan area, US state, practice size and patient characteristics (number of female, black and Hispanic patients, health risk score and dual public insurance). A provider was considered in a teaching location if an academic hospital was in the same ZIP code as the provider’s business ZIP code.

### Statistical analysis

Fixed-effects multiple ordinary least squares (OLS) regression models were fitted to provide estimates for the relationship between antibiotics rates and medical school. Four models were fitted for each outcome (days supplied, claims, days per claim and cost), controlling for the set of covariates explained above. Given non-linearity, experience squared was added to the model. Standard errors were clustered at the state level. To adjust for the large sample size, an alpha cut-off of 0.06 was used based on the good standardized value $\left(0.05/\surd (\frac{n}{100})\right)$.^15^

## Results

### Study characteristics

A total of 197 540 providers were included. The characteristics of providers, patients and claims are presented in Table 1. Mid-level providers comprised 41.5% of all prescribing providers. Only 5.7% of the providers were from the top-ranked medical schools [1, 35). The average experience after completing graduate school was 11.2 years (SD 9.9). Most providers were in metropolitan areas (84.7%), with the US South being the most common location (39.4%).

**Table 1.** Characteristics of providers, patients and antibiotic claims (FY2013–2021)

Characteristics	
Provider (*n* = 197 540)	
Gender	*n* (%)
Male	88 529 (44.8)
Female	109 011 (55.2)
Specialty	*n* (%)
EM	18 788 (9.5)
FM	40 930 (20.7)
Hospitalist	5105 (2.6)
IM	43 826 (22.2)
NP	50 906 (25.8)
PA	30 930 (15.7)
Student	7055 (3.6)
Academic location	*n* (%)
Yes	69 253 (35.1)
No	128 287 (64.9)
Graduation school ranking	*n* (%)
US MD [1, 35)	11 193 (5.7)
US MD [35, 58)	10 965 (5.5)
US MD [58, 85)	11 562 (5.9)
US MD [85,118]	6505 (3.3)
Unranked MD	11 043 (5.6)
Ranked DO	5608 (2.8)
Unranked DO	13 207 (6.7)
IMG MD	45 277 (22.9)
Ranked mid-level	6080 (3.1)
Unranked mid-level	76 100 (38.5)
	Mean (SD)
Years of professional experience	11.2 (9.9)
**Geography**	
Metropolitan area	*n* (%)
Yes	167 295 (84.7)
No	30 245 (15.3)
Region	*n* (%)
Northeast	34 569 (17.5)
Midwest	41 717 (21.1)
South	77 894 (39.4)
West	43 360 (21.9)
**Patients**	Mean(SD)
Beneficiaries per provider	219.1 (211.3)
Age (years)	70.0 (4.1)
% Female beneficiaries per provider	60.9 (8.9)
% Black beneficiaries per provider	15.8 (17.9)
% Hispanic beneficiaries per provider	10.3 (16.3)
% With dual insurance	35.9 (18.7)
Risk score	1.6 (0.6)
**Claims**	Mean(SD)
Total antibiotic days supplied per provider	1084 (1676)
Rate of antibiotic days supplied/ 100 beneficiaries per provider	341.1 (497.1)
Total antibiotic claims per provider	111.1 (154.9)
Rate of claims/100 beneficiaries per provider	29.4 (25.6)
Antibiotic days per claim	9.7 (6.5)
Antibiotic cost per provider	2094 (8287)
Rate of antibiotic cost/100 beneficiaries per provider	736 (6455)

MD, Doctor of Medicine; IMG, International Medical Graduates; DO, Doctor of Osteopathy; SD, standard deviation.

### Primary outcome

**Figure 1.**
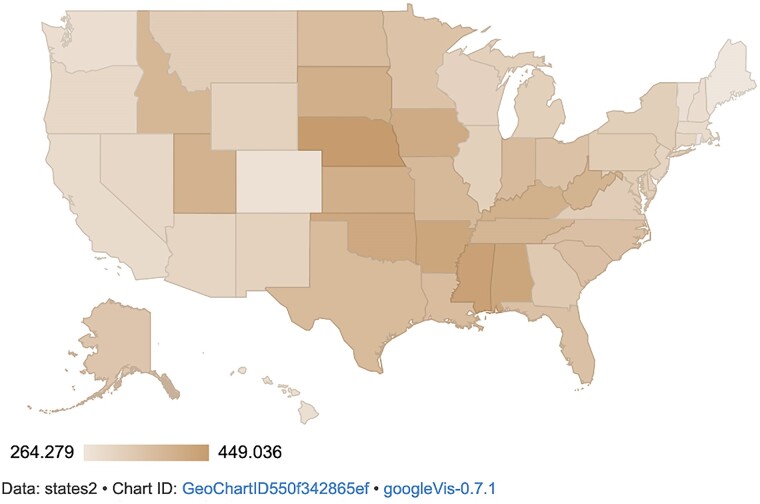
Antibiotic days supplied/100 beneficiaries per state (FY2013–2021). Based on CMS databases using the R program.

The average annual rate of antibiotic days supplied by a provider to 100 beneficiaries was 341.1 (SD 497.1) (Table 1). Figure 1 shows the distribution of days supplied per 100 beneficiaries across the USA. The South and Midwest had more days supplied than the Northeast and West. Nebraska had the highest rate (449.0), while Maine had the lowest (264.3).

**Figure 2.**
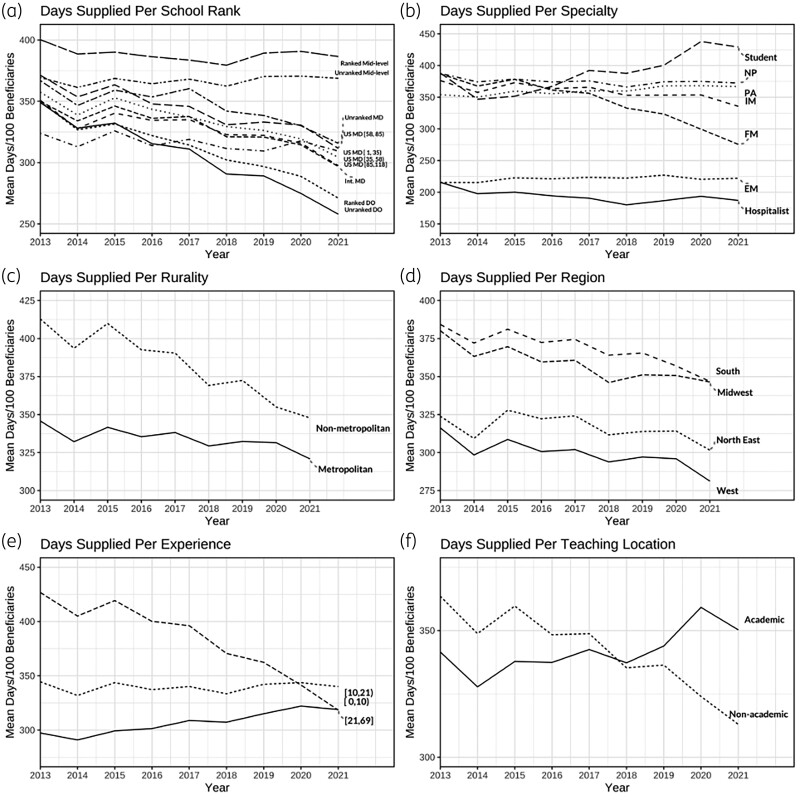
Antibiotic days supplied per 100 beneficiaries per category (FY2013–2021). Based on CMS databases using the R program.

Figure 2 shows the annual trend of days supplied per category. There was a general downtrend in the rate days supplied per 100 beneficiaries between 2013 and 2021 across most categories, except for mid-level providers, students and academic locations. Mid-level providers had the highest days supplied rate across school ranking, while DO providers had the lowest rate. Higher days supplied rate was seen among students, providers with more than 20 years of experience, providers in non-metropolitan areas and the South and Midwest.

### Secondary outcomes

The average annual claim rate per 100 beneficiaries was 111.1 (SD 154.9), and the average number of days per claim was 9.7 (SD 6.5) (Table 1). The distributions of claims rate and days per claim for each category are shown in Figures S1 and S2 (available as Supplementary data at *JAC-AMR* Online), respectively. Providers who graduated from the highest rank medical schools [1, 35) and providers in academic locations had the lowest claim rate per 100 beneficiaries but had more days per claim. Students had both more claims per 100 beneficiaries and more days per claim.

The total annual provider cost per 100 beneficiaries was USD 736.1 (SD 6455.1). Costs trended down across most categories between 2013 and 2017 and then increased afterward (Figure S3). Higher costs per 100 beneficiaries were seen among IMG, students, metropolitan areas, the US South and academic locations.

### Factors associated with study outcomes

Results from the multiple OLS regression model (Table 2) showed that medical school ranking, academic location and metropolitan area were not statistically significantly associated with the days supplied rate per 100 beneficiaries. Provider experience after graduate school was associated with more days supplied per 100 beneficiaries, though the relationship was parabolic, with a decreasing effect at the high range of experience. Students also had more days supplied per 100 beneficiaries. Male providers, EM providers and hospitalists had fewer days supplied per 100 beneficiaries than FM providers.

**Table 2.** OLS estimates for days supplied, rates of claims, days per claim and cost per 100 beneficiaries for FY2013–2021

	Supplied days rate estimate (SD)	Claim rate estimate (SD)	Days per claim estimate (SD)	Cost rate estimate (SD)
Education (Ref): US MD [1, 35]				
IMG MD	−22.50 (7.91)	3.60 (0.45)^a^	−1.76 (0.15)^a^	9.52 (42.70)
Ranked DO	−8.20 (8.25)	3.37 (0.38)^a^	−1.28 (0.11)^a^	30.43 (48.73)
Ranked mid-level	40.20 (21.18)	6.69 (1.10)^a^	−0.87 (0.28)	113.87 (131.93)
Unranked DO	−3.39 (8.98)	3.86 (0.58)^a^	−1.35 (0.11)^a^	29.53 (56.02)
Unranked MD	9.29 (7.53)	3.03 (0.41)^a^	−0.84 (0.08)^a^	65.56 (46.28)
Unranked mid-level	35.78 (18.19)	8.04 (1.00)^a^	−1.46 (0.24)^a^	164.32 (128.51)
US MD [35, 58]	−1.59 (8.83)	1.66 (0.51)	−0.50 (0.10)^a^	32.05 (38.81)
US MD [58, 85]	10.49 (8.10)	2.60 (0.45)^a^	−0.70 (0.10)^a^	52.04 (53.96)
US MD [85,118]	1.16 (9.63)	2.62 (0.67)^a^	−0.73 (0.11)^a^	34.76 (48.26)
Rural areas	26.15 (8.19)	3.22 (0.72)^a^	−0.22 (0.06)	−58.65 (26.84)
Male providers	−49.50 (4.77)^a^	2.33 (0.27)^a^	−1.46 (0.07)^a^	−130.83 (43.73)
Experience (years)^b^	9.34 (0.40)^a^	0.47 (0.03)^a^	0.12 (0.00)^a^	36.73 (5.26)^a^
Experience^2^b^	−0.07 (0.01)^a^	−0.00 (0.00)	−0.00 (0.00)	−0.53 (0.07)^a^
Specialty (Ref): FM				
EM	−130.74 (9.17)^a^	−5.76 (0.94)^a^	−2.31 (0.16)^a^	−528.55 (64.05)^a^
Hospitalist	−232.72 (13.10)^a^	−10.55 (0.88)^a^	−3.46 (0.17)^a^	−529.63 (104.34)^a^
IM	−16.50 (5.57)	−1.43 (0.39)	−0.07 (0.09)	186.45 (35.94)^a^
NP	26.24 (15.06)	−0.65 (0.97)	0.72 (0.23)	−23.14 (140.41)
PA	23.01 (15.45)	−0.82 (0.92)	0.81 (0.24)	−50.89 (135.54)
Student	99.74 (12.94)^a^	5.62 (0.76)^a^	1.36 (0.19)^a^	640.75 (107.65)^a^
Academic location	11.22 (4.04)	−1.48 (0.29)^a^	0.51 (0.06)^a^	112.34 (25.09)^a^

The model controlled for prescriber gender, specialty, graduation year, teaching location, metropolitan area, the US state and beneficiary characteristics (total number, demographics, risk scores and dual public insurance).

^a^ *P* value <0.0005.

^b^Experience was entered into the model as a continuous variable with a squared term given a non-linear relationship.

OLS, ordinary least squares regression; FY, fiscal year; SD, standard deviation; Ref, reference; MD, Doctor of Medicine; IMG, International Medical Graduates; DO, Doctor of Osteopathy

Higher-ranking medical schools [1, 35], EM providers and hospitalists (versus FM) and academic locations had lower claim rates per 100 beneficiaries, while students and experienced providers had higher claim rates. Days per claim were higher among providers from higher-ranked medical schools, more experienced providers, students and academic locations, whereas they were lower among males, EM providers and hospitalists. Costs per 100 beneficiaries were higher among students, academic locations, IM providers and males; however, it was lower among EM and hospitalists. Sensitivity analysis using (i) imputed values of 1, (ii) imputed values of 9, (iii) for FY2013 alone and (iv) for FY2019 alone showed similar results (data not shown).

## Discussion

Our study showed no impact of medical school ranking on the overall rate of outpatient antibiotic prescriptions among Medicare Part D providers. While the claim rate per 100 beneficiaries was lower among providers from higher-rank medical schools compared with other providers, claims were prescribed longer, leading to similar days supplied and costs compared with other providers. The academic location had a similar association with study outcomes. Students and more experienced providers had more claims per 100 beneficiaries, days per claim and costs per 100 beneficiaries. Conversely, EM and hospitalists had fewer claims per 100 beneficiaries, fewer days per claim and lower costs per 100 beneficiaries compared with FM.

We saw some reduction in the overall days supplied per 100 beneficiaries between FY2013 and 2021, driven mainly by a decrease in the claims rate per 100 beneficiaries. However, although national guidelines have shifted to recommend shorter duration for antibiotics for most conditions, ‘shorter is better’,^16^ an increase in the days per claim was demonstrated throughout most study groups. The cost of antibiotics per 100 beneficiaries has increased since 2018 due to a rise in the price of generics and the entrance of new brand inhaled anti-infective drugs to the US market (e.g. liposomal amikacin and aztreonam) (results not shown).

To our knowledge, this is the first study to assess the impact of medical school ranking on provider antimicrobial prescriptions and associated costs. Previous studies have shown conflicting results on the effects of medical school ranking on the quality of care in general. Tsugawa *et al*.^17^ showed no association between medical school ranking and patient mortality and readmission; however, higher medical school ranking was associated with lower healthcare spending among graduates. Schnell *et al*.^9^ showed lower opioid prescriptions among graduates of higher-ranking schools. Whether different priorities of medical school curricula could explain the difference in outcomes between opioid and antibiotic prescriptions among schools is unclear.

Other studies have demonstrated that medical schools^7,8^ varied in their AS training, including the number of students involved, methods of instruction, training duration and how programme outcomes were evaluated. Most AS interventions at the medical school or postgraduate level were either not assessed, or the evaluations focused solely on student reaction or learning, with very few determining whether there was a change in students’ or trainees’ behaviours or outcomes.^7^ These studies concluded that medical schools should implement robust evidence-based AS approaches with follow-up after training to ensure that skills are retained.

Similar to high-ranked medical schools, providers in academic locations had comparable days supplied per 100 beneficiaries, lower claim rates and higher days per claim compared with non-academic locations. In addition, they had higher costs per 100 beneficiaries. This was demonstrated despite having more robust AS programmes in academic hospitals.^18^

The absence of a difference in prescription rates between academic and non-academic locations could be explained by the focus of AS programmes on the inpatient rather than the outpatient setting. The emphasis on outpatient AS in the USA is relatively new, demonstrated by the CDC report on outpatient elements (published in 2016)^19^ and the Joint Commission requirements for AS in ambulatory healthcare (effective 2020).^20^ Currently, there are no CMS rules related to outpatient AS, and the Joint Commission requirements do not address outcome measures (e.g. change in prescription rates or appropriateness).

We found that hospitalists and EM had lower claim rates and days per claim than FM providers. These prescribers may only write a short course of therapy and defer longer courses or refills to primary care physicians (FM or IM). On the other hand, students and more experienced providers (>20 years) had higher claim rates and days per claim than other groups. It is possible that providers with less experience (≤20 years) are prescribing fewer days supplied due to learning about AS during their postgraduate training or through continuous medical education efforts. The term AS was only coined in 1996,^21^ and the more experienced providers may be less familiar with AS guidelines and efforts. Notably, the gap between experienced and less experienced providers has completely closed in 2021 (Figure 2). Conversely, days supplied and antibiotic-related costs associated with students continued to rise, largely due to increased days per claim, reflecting a great opportunity for future stewardship interventions. Notably, these prescriptions were most likely made under direct supervision and approval from licensed providers, which still presents a significant opportunity for targeted stewardship interventions during clinical training.

We hypothesized that graduating from higher-ranking medical schools was associated with better antimicrobial prescription due to potentially stronger training in AS. The US News and World Report for Best Medical Schools was used to categorize schools based on their research ranking for FY2023–2024. The report evaluated schools based on financial resources, new student achievements, qualitative evaluations and research productivity.^22^ While this ranking primarily emphasizes research funding and academic output, they do not include specific factors related to the Infectious Diseases curriculum or AS training. However, higher-ranked schools often have more resources and infrastructure to offer comprehensive clinical training, which could contribute to better stewardship. Although we anticipated that such advantages might translate into improved prescribing patterns, our findings indicate that these potential benefits have not had a sustained impact on antibiotic prescription behaviours among graduates.

We used the FY2023–2024 school ranking rather than the year the provider graduated, as we could not access the older rankings. It is important to note that the US News and World Report has been criticized for poor design, elitist views and lack of outcome assessment from quality perspectives.^23^ However, alternative metrics are lacking to evaluate medical schools based on education quality, clinical care outcomes, research impact and community benefit.^23^

Our study has several limitations. First, we could not assess differences in the curriculum or training related to AS or infectious diseases across medical schools, as there are no standardized data on curricular content and educational outcomes in the USA. There was likely significant variation over time and differences in the quality, quantity and format of AS and infectious diseases education across medical schools. Current medical education guidelines in the USA emphasize competency-based training that includes didactic sessions, clinical experiences and assessments of knowledge in AS and infectious diseases. However, implementing these outcomes varies widely between institutions, making it challenging to determine their impact on prescribing behaviours in our analysis.

A second limitation is that we were unable to assess antibiotic appropriateness as we did not have access to the antimicrobial indication (e.g. upper respiratory infection) or other clinical characteristics. We also used the presence of a teaching hospital in the same ZIP code as a surrogate of academic affiliation. This approach has been previously used in the literature^9^; however, we have likely overestimated the number of academic providers as their presence in a ZIP code with a teaching hospital does not necessarily reflect an academic affiliation.

Regarding the medical school ranking, we placed all IMG in one category as we did not have access to the country of the IMG medical school to evaluate its international ranking. Additionally, most mid-level and DO providers graduated from unranked medical schools, making it challenging to assess the role of DO and mid-level school ranking on antibiotic prescription rates. It is also important to note that medical school rankings primarily reflect research productivity rather than educational outcomes related to AS training. Furthermore, we did not account for the influence of postgraduate education, which likely plays a significant role in prescribing practices. Lastly, this study had an ecological design, limiting the ability to make a temporal link between independent variables and study outcomes.

One key strength of this study is that it has assessed antibiotic utilization nationwide for almost a decade. This allows generalizability to general practitioners in the USA. In addition, we evaluated several metrics of antibiotic prescription (days supplied, claims rate, days per claim and cost) and adjusted per the number of beneficiaries to account for practice size. Third, we utilized multiple OLS regression models and adjusted for several variables (e.g. demographics, risk score, dual insurance and US state) that could confound the relationship between the independent and dependent variables. Finally, given the large repository size, we used a standardized *P* value of 0.0005 rather than 0.05, limiting Type I error.

Our study provides vital information to public health professionals. It demonstrates an improvement in claim rates (2013–2021) but highlights that days per claim remain an opportunity for outpatient stewardship efforts in older patients. These findings allow antibiotic stewards to target outpatient educational efforts to students. It also shows a need to create more robust AS programmes and medical school training that focus on improving prescription rates in the outpatient setting.

In conclusion, our study revealed that medical school ranking did not impact the rate of antibiotic days supplied per 100 beneficiaries. Students and providers with over 20 years of experience had the highest days supplied rate. AS interventions should target students and build outcome-based medical school AS training that prioritizes reducing the antimicrobial duration of therapy.

## Supplementary Material

dlae191_Supplementary_Data

## Supplementary data

Figures S1–S3 are available as Supplementary data at *JAC-AMR* Online.

## References

[1] Centers for Disease Control and Prevention . Antibiotic Resistance Threats in the United States 2019. https://www.cdc.gov/antimicrobial-resistance/media/pdfs/2019-ar-threats-report-508.pdf?CDC_AAref_Val=https://www.cdc.gov/drugresistance/pdf/threats-report/2019-ar-threats-report-508.pdf

[2] O’Neil J. Antimicrobial Resistance: Tackling A Crisis for the Health and Wealth of Nations. https://amr-review.org/sites/default/files/AMR%20Review%20Paper%20-%20Tackling%20a%20crisis%20for%20the%20health%20and%20wealth%20of%20nations_1.pdf

[3] Gouin KA, Fleming-Dutra KE, Tsay S, et al Identifying higher-volume antibiotic outpatient prescribers using publicly available Medicare part D data—United States, 2019. MMWR 2022; 71: 202–5. 10.15585/mmwr.mm7106a3 35143465 PMC8830623

[4] Kim C, Kabbani S, Dube WC et al Health equity and antibiotic prescribing in the United States: a systematic scoping review. Open Forum Infect Dis 2023; 10: ofad440. 10.1093/ofid/ofad440 37671088 PMC10475752

[5] McKay R, Mah A, Law MR et al Systematic review of factors associated with antibiotic prescribing for respiratory tract infections. Antimicrob Agents Chemother 2016; 60: 4106–18. 10.1128/AAC.00209-16 27139474 PMC4914667

[6] Schmiege D, Evers M, Kistemann T et al What drives antibiotic use in the community? A systematic review of determinants in the human outpatient sector. Int J Hyg Environ Health 2020; 226: 113497. 10.1016/j.ijheh.2020.113497 32120251

[7] Augie BM, Miot J, van Zyl RL et al Educational antimicrobial stewardship programs in medical schools: a scoping review. JBI Evid Synth 2021; 19: 2906–28. 10.11124/JBIES-20-00330 34392265

[8] Silverberg SL, Zannella VE, Countryman D et al A review of antimicrobial stewardship training in medical education. Int J Med Educ 2017; 8: 353–74. 10.5116/ijme.59ba.2d47 29035872 PMC5694692

[9] Schnell M, Currie J. Addressing the opioid epidemic: is there a role for physician education? American journal of health economics 2018; 3: 383–410. 10.1162/ajhe_a_00113 PMC6258178 30498764

[10] Centers for Medicare and Medicaid Services . Medicare Part D Prescribers—by Provider. https://data.cms.gov/provider-summary-by-type-of-service/medicare-part-d-prescribers/medicare-part-d-prescribers-by-provider

[11] Centers for Medicare and Medicaid Services . Medicare Part D Prescribers—by Provider and Drug. https://data.cms.gov/provider-summary-by-type-of-service/medicare-part-d-prescribers/medicare-part-d-prescribers-by-provider-and-drug/data

[12] Centers for Medicare and Medicaid Services . National Downloadable File. https://data.cms.gov/provider-data/dataset/mj5m-pzi6

[13] Centers for Medicare and Medicaid Services . Open Payments List of Teaching Hospitals. https://www.cms.gov/files/document/open-payments-2023-report-cycle-teaching-hospital-listp.pdf

[14] U.S. News & World Report . 2023–2024 Best Medical Schools: Research. https://www.usnews.com/best-graduate-schools/top-medical-schools/research-rankings

[15] Beers B. P-Value: What It Is, How to Calculate It, and Why It Matters. https://www.investopedia.com/terms/p/p-value.asp#:∼:text=A%20p%2Dvalue%20of%200.05,confidence%20levels%20for%20hypothesis%20testing

[16] Grant J, Saux NL. Antimicrobial Stewardship and Resistance Committee (ASRC) of the Association of Medical Microbiology and Infectious Disease (AMMI) Canada. Duration of antibiotic therapy for common infections. J Assoc Med Microbiol Infect Dis Can 2021; 6: 181–97. 10.3138/jammi-2021-04-29 36337760 PMC9615468

[17] Tsugawa Y, Blumenthal DM, Jha AK et al Association between physician US News & World Report medical school ranking and patient outcomes and costs of care: observational study. BMJ 2018; 362: k3640. 10.1136/bmj.k3640 30257919 PMC6156557

[18] Barlam TF, DiVall M. Antibiotic-stewardship practices at top academic centers throughout the United States and at hospitals throughout Massachusetts. Infect Control Hosp Epidemiol 2006; 27: 695–703. 10.1086/503346 16807844

[19] Centers for Disease Control and Prevention . Core Elements of Outpatient Antibiotic Stewardship. https://www.cdc.gov/antibiotic-use/hcp/core-elements/outpatient-antibiotic-stewardship.html?CDC_AAref_Val=https://www.cdc.gov/antibiotic-use/core-elements/outpatient.html

[20] The Joint Commission . R3 Report Issue 23: Antimicrobial Stewardship in Ambulatory Health Care. https://www.jointcommission.org/standards/r3-report/r3-report-issue-23-antimicrobial-stewardship-in-ambulatory-health-care/

[21] McGowan JE JR, Gerding DN. Does antibiotic restriction prevent resistance? New Horiz 1996; 4: 370–6. 8856755

[22] U.S. News & World Report . Methodology: 2023–2024 Best Medical Schools Rankings. https://www.usnews.com/education/best-graduate-schools/articles/medical-schools-methodology

[23] McGaghie WC . America’s best medical schools: a renewed critique of the US news & world report rankings. Acad Med 2019; 94: 1264–6. doi:10.1097/ACM.0000000000002742 31460911

