# Supplementary material for: Medical school ranking and provider outpatient Medicare Part D claims for antibiotics among older patients in the USA
Source: JAC Antimicrob Resist. 2024 Nov 23;6(6):dlae191. doi: 10.1093/jacamr/dlae191 (PMC11584510; doi:10.1093/jacamr/dlae191)
Supplement: dlae191_Supplementary_Data [file dlae191_supplementary_data.docx]

**Supplemental Materials**

**Supplement Figure 1- Antibiotic Claims Per 100 Beneficiaries Per Category (FY2013-2021)**


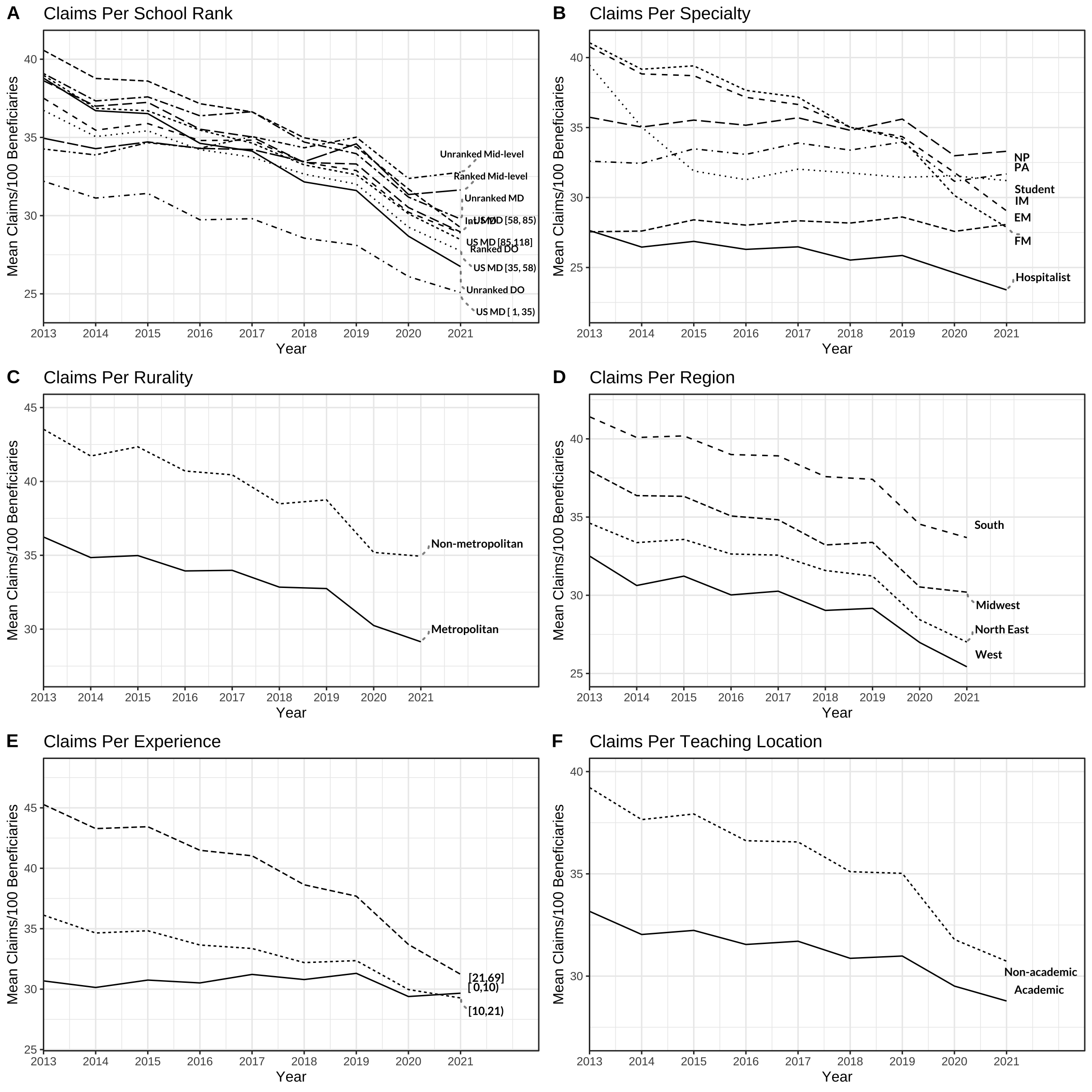


This figure shows antibiotic claims per 100 beneficiaries per category, grouped by graduate school ranking (A), Specialty (B), Metropolitan Area (C), Region (D), years of experience (E), and teaching location (F).

MD: Doctor of Medicine; Int: International Medical Graduate; DO: Doctor of Osteopathy; NP: Nurse Practitioner; PA: Physician Assistant; IM: Internal Medicine; FM: Family Medicine: EM: Emergency Medicine

**Supplement Figure 2- Antibiotic Days per Claim Per Category (FY2013-2021)**


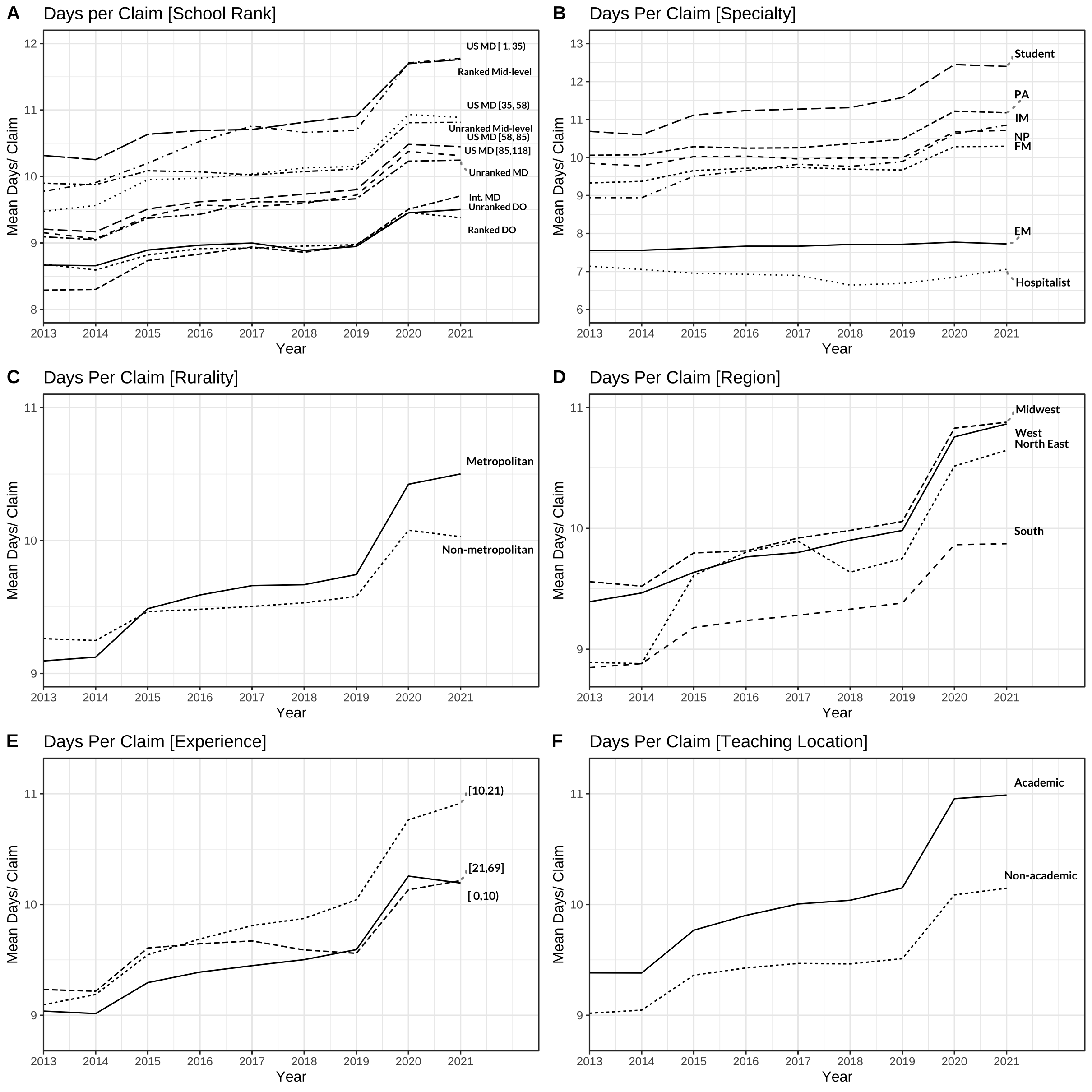


This figure shows days per claim per category, grouped by graduate school ranking (A), Specialty (B), Metropolitan Area (C), Region (D), years of experience (E), and teaching location (F).

MD: Doctor of Medicine; Int: International Medical Graduate; DO: Doctor of Osteopathy; NP: Nurse Practitioner; PA: Physician Assistant; IM: Internal Medicine; FM: Family Medicine: EM: Emergency Medicine

**Supplement Figure 3- Antibiotic Cost Per 100 Beneficiaries Per Category (FY2013-2021)**


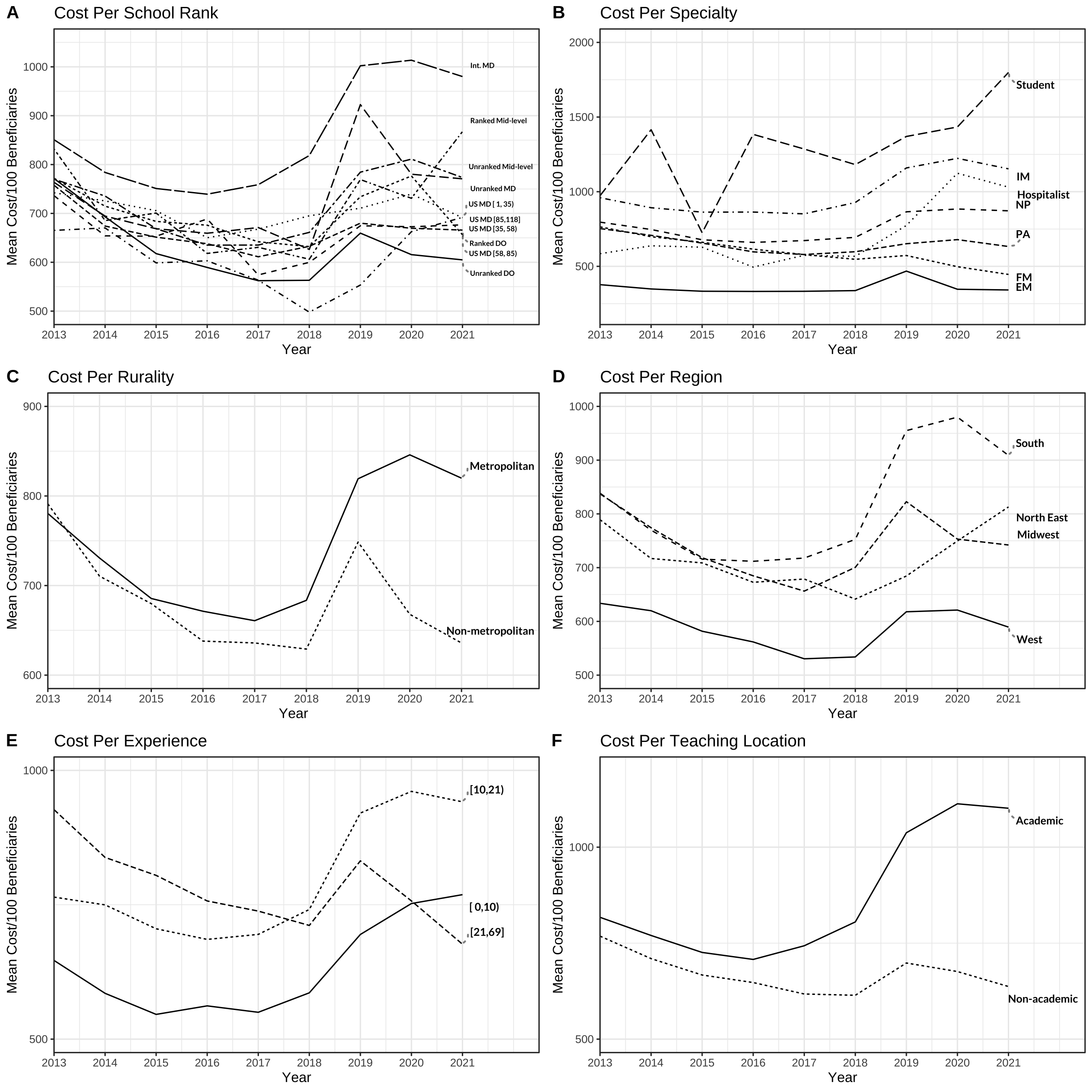


This figure shows the antibiotic cost per 100 beneficiaries per category, grouped by graduate school ranking (A), Specialty (B), Metropolitan Area (C), Region (D), years of experience (E), and teaching location (F).

MD: Doctor of Medicine; Int: International Medical Graduate; DO: Doctor of Osteopathy; NP: Nurse Practitioner; PA: Physician Assistant; IM: Internal Medicine; FM: Family Medicine: EM: Emergency Medicine
